# VarPPUD: Pinpointing diagnostic variants from sets of prioritized, strong candidate variants

**DOI:** 10.1371/journal.pcbi.1013414

**Published:** 2025-09-22

**Authors:** Rui Yin, Alba Gutiérrez-Sacristán, Undiagnosed Diseases Network, Shilpa Nadimpalli Kobren, Paul Avillach

**Affiliations:** 1 Department of Biomedical Informatics, Harvard Medical School, Boston, Massachusetts, United States of America; 2 Department of Health Outcomes and Biomedical Informatics, University of Florida, Gainesville, Florida, United States of America; Washington University in Saint Louis, UNITED STATES OF AMERICA

## Abstract

Rare and ultra-rare genetic conditions are estimated to impact nearly 1 in 17 people worldwide, yet accurately pinpointing the diagnostic variants underlying each of these conditions remains a formidable challenge. Because comprehensive, *in vivo* functional assessment of all possible genetic variants is infeasible, clinicians instead consider *in silico* variant pathogenicity predictions to distinguish plausibly disease-causing from benign variants across the genome. However, in the most difficult undiagnosed cases, such as those accepted to the Undiagnosed Diseases Network (UDN), existing pathogenicity predictions cannot reliably discern true etiological variant(s) from other deleterious candidate variants that were prioritized through case- or family-level analyses. Pinpointing the disease-causing variant from a small pool of plausible candidates remains a largely manual effort requiring extensive clinical workups, functional and experimental assays, and eventual identification of genotype- and phenotype-matched individuals. Here, we introduce VarPPUD, a tool trained on prioritized variants from UDN cases, that leverages gene-, amino acid-, and nucleotide-level features to discern pathogenic (disease causative) variants from other damaging or deleterious variants that are unlikely to be confirmed as relevant to the disease. VarPPUD achieves a cross-validated accuracy of 79.3% and precision of 77.5% on a held-out subset of uniquely challenging UDN cases, respectively representing an average 18.6% and 23.4% improvement over nine existing state-of-the-art pathogenicity prediction tools on this task. We validate VarPPUD’s ability to discriminate likely from unlikely pathogenic variants using both synthetic data generated via a GAN-based framework and a temporally held-out set of UDN patients evaluated between 2022 and 2024. The model was trained exclusively on data available through 2021 and applied without retraining to the post-2021 cohort, demonstrating strong generalizability to newly accrued cases. Finally, we show how VarPPUD can be probed to evaluate each input feature’s importance and contribution toward prediction—an essential step toward understanding the distinct characteristics of newly-uncovered disease-causing variants.

## Author summary

Patients with chronic, undiagnosed and underdiagnosed genetic conditions often endure expensive and excruciating years-long diagnostic odysseys without clear results. In many instances, clinical genome sequencing of patients and their family members fails to reveal known disease-causing variants, although compelling variants of uncertain significance are frequently encountered. Existing computational tools struggle to reliably differentiate truly disease-causing variants from other plausible candidate variants within these prioritized sets. Consequently, the confirmation of disease-causing variants often necessitates extensive experimental follow-up, including studies in model organisms and identification of other similarly presenting genotype-matched individuals, a process that can extend for several years. Here, we present VarPPUD, a tool trained specifically to distinguish likely from unlikely to be confirmed pathogenic variants that were prioritized across cases in the Undiagnosed Diseases Network. By evaluating the importance and impact of different input feature values on prediction, we gain deeper insights into the distinctive attributes of difficult-to-identify diagnostic variants. For patients who remain undiagnosed following comprehensive whole genome sequencing, our new method VarPPUD may reveal pathogenic variants amid a pool of strong candidate variants, thereby advancing diagnostic efforts where progress has otherwise stalled.

## Introduction

Rare and undiagnosed suspected genetic conditions cumulatively affect an estimated 25–30 million Americans and millions more worldwide [1]. These diseases are typically associated with premature mortality or lifelong disability [2,3]. However, each specific genetic condition may impact only a handful of individuals or as few as a single individual or family, hindering traditional case-control statistical approaches to study disease. As a result, diagnosing these conditions and uncovering disease-causing variants and mechanisms of action is extremely difficult. Indeed, patients suffering from rare disorders can spend multiple years and hundreds of thousands of dollars in pursuit of a genetic diagnosis [4]. Despite a steady increase in the identification of genetic aberrations that underlie rare Mendelian disorders, many conditions remain undiagnosed, in large part because patients’ presenting symptoms and disease-causing variants do not match known diseases.

To facilitate the diagnosis of rare and novel genetic disorders and further our collective understanding of the pathogenesis of these diseases, the United States’ National Institutes of Health funded the Undiagnosed Diseases Network (UDN) in 2014 [5–7]. Patients who are eventually referred to and accepted into the UDN undergo an extensive clinical evaluation with medical specialists at one of 15+ clinical research sites across the country [8]. The causes of undiagnosed disease are multifaceted, with genetic variants considered a primary factor [9]. As such, in a majority of cases, UDN patients and their relevant affected and unaffected family members also receive whole genome sequencing. This data is computationally analyzed and filtered to select for variants that are sufficiently rare in control populations, inherited in appropriate patterns, and have high functional impacts on phenotypically-relevant genes [10]. Even after this process, not all prioritized variants are causal, and indeed most are eventually found—through arduous manual investigation and experimentation—to have no impact on health. Improved computational discernment of candidate variants that are eventually confirmed *pathogenic*, or disease causal, is therefore of utmost importance.

Several computational tools have been developed to differentiate deleterious and pathogenic variants from benign variants in various disease contexts [11–13]. These *in silico* approaches have utilized diverse features based on amino acid sequence and protein structure (e.g., PolyPhen2 [14]), evolutionary conservation (e.g., MutationAssessor [15], PROVEAN [16]), and phenotypic and other biomedical associations (e.g., SIFT [17], MutationTaster [18,19]). Various underlying prediction models have also been explored, including hidden Markov models (e.g., FATHMM [20]), supervised machine learning (e.g., VEST [21]) and ensemble approaches (e.g., REVEL [22], CADD [23,24]). These algorithms have been utilized extensively and successfully to prioritize disease-causing variants of uncertain significance (VUS) in clinical contexts, demonstrating the utility of predictive algorithms in diagnostic medicine. Nevertheless, given a set of rare, functional, and clinically plausible candidate variants uncovered in an undiagnosed patient, existing pathogenicity predictors cannot easily distinguish disease-causing from non-causal or unlikely to be confirmed causal variants. Moreover, models underlying existing pathogenicity predictors tend to abstract away the contribution of each individual input feature when producing a final classification or score, making prediction interpretability and determination of variants’ functional impacts more difficult.

Here, we present VarPPUD (**V**ariant **P**ost **P**rioritization for **U**ndiagnosed **D**isorders), a random forest-based model that classifies VUS as likely- or unlikely-to-be confirmed pathogenic based on gene-level functionality and evolutionary constraint, physicochemical properties of amino acids, and existing nucleotide-level variant deleteriousness predictions. VarPPUD is trained on a uniquely challenging set of real-world diagnostic and candidate variants identified in UDN patients through 2021 uncovered by extensive manual efforts by clinical experts. In contrast to other publicly-accessible databases with disease-associated genetic variants (e.g., ClinVar [25], HGMD [26]), which are limited primarily to confirmed diagnostic variants or other variants in genes already known to be associated with disease, the UDN dataset contains especially difficult-to-interpret variants with no restrictions on preexisting gene associations or variant type. Unlike other rare disease cohorts [27], the UDN accepts patients with both childhood- and adult-onset conditions that span a wide range of primary symptoms. A predictor trained on causal variants from this cohort would thus be generalizable across disease contexts. We show that VarPPUD is better able to discern likely pathogenic from other candidate variants relative to existing methods on a held-out set of this real-world dataset. We also show that VarPPUD can generalize well to new cases, as demonstrated by its performance on a temporally distinct dataset derived from a newer cohort of UDN patients enrolled between 2022 and 2024. To address the issue of small sample sizes of candidate variants in real-world UDN patients, we further validate VarPPUD’s performance on a synthetic dataset produced via a Generative Adversarial Network (GAN) [28–33]. GANs offer several compelling advantages in our context. Unlike synthetic oversampling approaches, GANs can capture joint distributions and non-linear relationships between features and are amenable to enforcing value constraints [34]. Additionally, unlike basic resampling methods (e.g., bootstrapping, permutation), GANs produce entirely new samples, which is crucial to avoid overfitting to limited examples. Finally, VarPPUD’s underlying framework enables us to use Shapley Additive Explanation values [35] to probe the contribution of each input feature and identify, for each prediction, the specific lines of evidence used to designate a candidate variant as likely pathogenic or not.

## Materials and methods

### Variant compilation and pathogenicity classification

**Fig 1:**
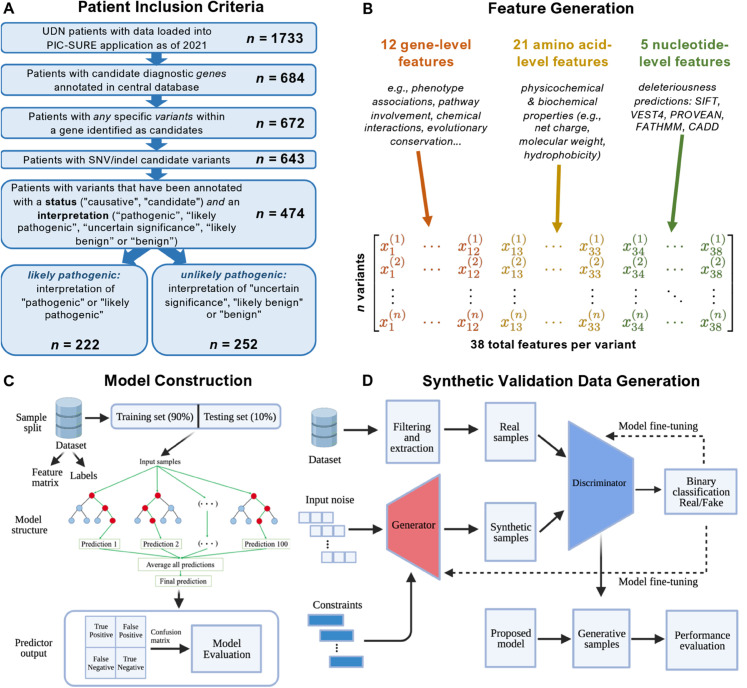
VarPPUD construction and validation process. A: Procedure for patient inclusion in our study. UDN patients lacking information about candidate genes, variants responsible for undiagnosed disorders, and unknown variant interpretations were excluded. Patients with long insertion/deletion (>5 nucleotides) or complicated variants on candidate genes were also filtered from the cohort. B: Feature generation. We leveraged three categories i.e., gene-level functionality and evolutionary constraint, physicochemical properties of amino acids, and existing variant deleteriousness predictions, for feature generation. These features were acquired using various databases and tools based on the category of input data. The variables generated through distinct approaches were concatenated into a matrix and fed into machine learning models. C: Model construction. Filtered and processed samples were divided into training (90%) and testing (10%) datasets. Five-fold cross-validation was performed on the training set with a random forest classifier. The final performance estimated for the VarPPUD was quantified by metrics such as accuracy, precision, and recall on the held-out testing test. D: GAN-based validation. To validate the robustness of VarPPUD on new undiagnosed cases, we constructed a constraint-based generative adversarial network to produce synthetic testing data. We used the area under the Receiver Operator Characteristic (ROC) and Precision-Recall (PR) curves to evaluate the model’s performance.

Upon acceptance to the UDN, each patient is assigned to one of 15+ clinical sites where a team of medical, genetic, and bioinformatic specialists works collaboratively to find a diagnosis. These teams perform extensive in-person clinical evaluations of affected patients and relevant family members and analyze patients’ medical records, clinical reports, and prior or newly requested sequencing data to identify candidate disease-causing genetic variants [10]. Computationally-prioritized variants are often manually reviewed for conconcordance with a patient’s phenotype, and clinical teams upload any remaining, few strong candidate genetic variants to the UDN Data Management and Coordinating Center and annotate them with a status of “causative”, “candidate”, or “rejected” and an interpretation of “pathogenic”, “likely pathogenic”, “uncertain significance”, “likely benign” or “benign” as recommended by the College of American Pathologists [36,37]. All per-patient prioritized variants, standardized phenotype terms, demographic information and clinical data as of 2021 were subsequently loaded into a PIC-SURE instance for fast multi-modal querying (https://avillach-lab.hms.harvard.edu/pic-sure). Note that only confirmed pathogenic or highly likely pathogenic variants are submitted to ClinVar, whereas other strong candidate variants with uncertain significance or which are eventually rejected may not be submitted to ClinVar [25]. From the 1733 patients evaluated in the UDN as of December 2021, we selected 474 patients with annotated SNV/indel candidate variants with a status of “causative” or “candidate” and an available pathogenicity interpretation (Fig 1A). Each patient only had exactly one variant prioritized for a total of 474 variants considered. Population frequency analyses suggest that most variants annotated as “uncertain significance” are likely benign [37]. Although some VUS may be disease causal, confirming their pathogenicity may be prohibitively difficult given a gene’s lack of model organism orthologs, patient’s exhibited phenotypes, and/or variant’s functional impact. We therefore reclassified all variants into two groups: 222 “likely pathogenic” variants with an original interpretation of “pathogenic” or “likely pathogenic”, and 252 “unlikely pathogenic” variants with an original interpretation of “uncertain significance”, “likely benign” or “benign”. Note that we use the phrase “unlikely pathogenic” to encompass both variants that are truly unlikely to be pathogenic as well as variants that are unlikely to be confirmed pathogenic in a short amount of time. Patient demographics grouped by prioritized variant category can be found in Table 1.

**Table 1:** Demographic and clinical characteristics of patients grouped by the category of their prioritized variant.

Category		Missing	*Overall*	*Weakly Pathogenic*	*Strongly Pathogenic*
Number of patients, n		-	474	252	222
Age in years at evaluation, mean (SD)		14	15.0 (15.6)	15.4 (16.1)	14.6 (15.0)
Age in years at symptom onset, mean (SD)		-	6.1 (13.3)	6.7 (13.7)	5.5 (12.8)
*Ethnicity, n (%)*	Hispanic or Latino	1	88 (18.6)	47 (18.7)	41 (18.6)
	Not Hispanic or Latino		351 (74.2)	188 (74.6)	163 (73.8)
	Unknown or Not Reported		34 (7.2)	17 (6.7)	17 (7.7)
*Gender, n (%)*	Female	-	232 (48.9)	127 (50.4)	105 (47.3)
	Male		242 (51.1)	125 (49.6)	117 (52.7)
*Race, n (%)*	American Indian/Alaska Native	6	5 (1.1)	2 (0.8)	3 (1.4)
	Asian		42 (9.0)	19 (7.7)	23 (10.5)
	Black or African American		26 (5.6)	13 (5.2)	13 (5.9)
	Other		39 (8.3)	24 (9.7)	15 (6.8)
	White		356 (76.1)	190 (76.6)	166 (75.5)
*Primary symptom category, n (%)*	Allergies / disorders of the immune system	7	21 (4.5)	16 (6.5)	5 (2.3)
	Cardiology and vascular conditions		16 (3.4)	11 (4.4)	5 (2.3)
	Dentistry & craniofacial		2 (0.4)	2 (0.8)	-
	Dermatology		5 (1.1)	2 (0.8)	3 (1.4)
	Endocrinology		12 (2.6)	5 (2.0)	7 (3.2)
	Gastroenterology		20 (4.3)	14 (5.6)	6 (2.7)
	Gynecology & reproductive		1 (0.2)	1 (0.4)	-
	Hematology		5 (1.1)	2 (0.8)	3 (1.4)
	Musculoskeletal / orthopedics		72 (15.4)	34 (13.7)	38 (17.4)
	Nephrology		5 (1.1)	2 (0.8)	3 (1.4)
	Neurology		225 (48.2)	107 (43.1)	118 (53.9)
	Oncology		2 (0.4)	2 (0.8)	-
	Ophthalmology		9 (1.9)	3 (1.2)	6 (2.7)
	Other		50 (10.7)	31 (12.5)	19 (8.7)
	Psychiatry		2 (0.4)	1 (0.4)	1 (0.5)
	Pulmonology		10 (2.1)	5 (2.0)	5 (2.3)
	Rheumatology		10 (2.1)	10 (4.0)	-

Prioritized genetic variants are assigned to either the “weakly pathogenic” or “strongly pathogenic” categories based on their provided clinical interpretation. Each prioritized variant corresponds to a single affected patient.

### Gene-level features

We generated 12 gene-level features for each of the 474 variants in our real-world UDN dataset. These include: (1) number of unique phenotype terms associated with the gene from the Human Phenotype Ontology (HPO, https://hpo.jax.org/app/) [38], (2) number of pathways that the gene appears in as listed in the Comparative Toxicogenomics Database (CTD, http://ctdbase.org/) [39], (3) number of unique chemicals known to interact with the gene from CTD, (4) number of specific interactions between the gene and any chemical (i.e., chemical A inhibits the enzyme reaction of gene X, chemical A also reduces the expression of gene X) from CTD, (5) number of unique ways in which any chemical can interact with the gene (e.g., increases expression, affects folding, decreases reaction) from CTD, (6) number of rare diseases associated with the gene as listed in GeneAnalytics (https://geneanalytics.genecards.org/) [40], (7) number of total diseases associated with the gene from GeneAnalytics, (8) evolutionary age of the gene from ProteinHistorian (https://proteinhistorian.docpollard.org/) [41], (9) number of non-human species with an ortholog to the gene from phylogenetic profiles [42] in ProteinHistorian, (10) dN/dS scores indicating selective direction and pressure from Evola (http://www.h-invitational.jp/evola/search.html) [43], (11) essentiality scores indicating impact if both gene copies are lost from OGEE (https://v3.ogee.info/#/home) [44], and (12) haploinsufficiency scores indicating impact if only one gene copy is lost [45].

### Amino acid-level features

We selected 21 continuous-valued physicochemical and biochemical properties defined for each amino acid type (e.g., polarity, molecular weight) from AAindex (https://www.genome.jp/aaindex/, S1 Table) [46]. We determined the reference and alternate amino acid(s) for each prioritized variant wherever possible by retrieving their Human Genome Variation Society (HGVS) protein nomenclatures from LOVD (https://databases.lovd.nl/shared/variants) and MyGene2 (https://mygene2.org/MyGene2/genes).

For each amino acid property *x*, we compute an amino-acid based feature as $\Delta x(ref,alt)=f_{x}(alt)--f_{x}(ref)$, where *f*_*x*_ is the value of property *x* for the query amino acid, *ref* is the reference amino acid sequence, and *alt* is the alternate amino acid sequence. We define *f*_*x*_(*stop*) = 0 where *stop* is a termination codon and $f_{x}(A,B...,Z)=f_{x}(A)+f_{x}(B)+...+f_{x}(Z)$ where *A*, *B*, and *Z* are individual amino acids. For frameshift variants, we set the reference amino acids to be the original protein sequence from the position of the first changed amino acid through the original termination codon, and the alternate amino acids to be the new sequence from the position of the first changed amino acid through the new termination codon. We did not encounter any variants that resulted in the loss of a termination codon, but for completeness for this case, we consider up to twenty amino acids past the original termination codon to be the alternate amino acids.

### Nucleotide-level features

For each prioritized variant, we queried its impacted gene, transcript, and annotated cDNA change in LOVD, MyGene2 and ClinVar (https://www.ncbi.nlm.nih.gov/clinvar/submitters/505999/) to determine the variant’s chromosome, genomic position, reference nucleotide(s) and alternate nucleotide(s) with respect to human genome build hg19/GRCh37. With variants in this format, we then computed deleteriousness or pathogenicity prediction scores for five other methods—SIFT (https://sift.bii.a-star.edu.sg/) [17], VEST4 (http://cravat.us/CRAVAT/) [21], PROVEAN (http://provean.jcvi.org/index.php) [16], FATHMM (http://fathmm.biocompute.org.uk/) [20] and CADD (https://cadd.gs.washington.edu/) [23,24]—using their default, recommended parameters. Note that the first two predictors score only missense variants, PROVEAN also scores in-frame indels, and the last two predictors additionally score frameshift indels, nonsense and non-coding splice-altering variants. We did not include population allele frequency as a nucleotide-level feature because all UDN-prioritized variants had already been filtered for rarity. As such, this feature would be redundant and uninformative for distinguishing likely from unlikely pathogenic variants in this context. By definition, variants prioritized for ultrarare genetic conditions should be absent or exceedingly rare in population controls [10].

### Training the random forest classifier

We randomly split our 222 likely pathogenic and 252 unlikely pathogenic prioritized variants into training and validation sets in a 9:1 ratio, preserving the relative proportion of each variant type across the two sets. We then used Python’s Scikit-learn package [47] to train a random forest classifier in the training set using 5-fold cross-validation with bootstrapping to optimize model hyperparameters and reduce overfitting. Specifically, we randomly sampled with replacement from the training data to generate five folds—each with equivalent numbers of strongly and weakly pathogenic prioritized variants—and used each fold in turn as a held-out test set while training the model on the remaining four folds; we repeated this process 10 times. We also specified that each decision tree in the random forest model considered at most seven input features, minimized Gini impurity for each node split, had a maximum depth of five, had internal nodes with at least 30 samples, and had leaf nodes with at least 10 samples.

### Imputing missing feature values

Values for the 12 gene-based, 21 amino acid-based and 5 nucleotide-based features are missing in up to 37.6% of prioritized variants (Table 2). Because missing feature values may cause performance degradation in predictive models, we utilize Multivariate/Multiple Imputation in Chained Equations (MICE) to fill these in [48,49]. Briefly, missing values for each feature are initialized with the mean of the non-missing values for that feature. Then, for each feature at a time, we fit a linear regressor using all other features as input (utilizing both the true observed and filled-in missing values) and the selected feature as output. We apply this fitted regressor to predict and fill in missing values for the selected feature. We then iteratively repeat this process over all features with missing values until convergence where values are no longer updated using the LinearRegression and IterativeImputer functions in Python’s Scikit-learn package. Imputing missing feature values boosted VarPPUD’s classification accuracy by 18.6%, precision (with respect to likely pathogenic variants) by 23.4%, and F-score by 14.5% during five-fold cross validation on the training set. Imputing missing values improved the performance of VarPPUD on the held-out validation set as well (Table 3). The final VarPPUD random forest model trained with imputed feature values had 340 individual decision trees.

**Table 2:** Feature value properties for UDN-prioritized variants.

Type	Feature	Range	Missing	Mean	Standard Deviation
*gene-based*	# phenotypes associated with gene	{0 .. +∞}	0 (0%)	172	169
	# pathways gene is part of	{0 .. +∞}	0 (0%)	13.7	25.8
	# unique chemicals gene interacts with	{0 .. +∞}	0 (0%)	36.4	34.2
	# total chemical-gene interactions	{0 .. +∞}	0 (0%)	47.1	66.9
	# chemical-gene interaction types	{0 .. +∞}	0 (0%)	11.4	10.6
	# rare diseases associated with gene	{0 .. +∞}	0 (0%)	21.3	24.4
	# total diseases associated with gene	{0 .. +∞}	0 (0%)	30	36.9
	evolutionary age	[0, +∞)	42 (8.9%)	989	837
	# species with an ortholog	{0 .. +∞}	42 (8.9%)	2.51	3.07
	dN/dS	[0, 1]	68 (14.3%)	0.18	0.162
	gene essentiality	{0 .. 2}	45 (9.5%)	0.455	0.543
	gene haploinsufficiency	[0, 1]	142 (30.0%)	0.496	0.251
*amino acid-based*	polarity	[–2.54, 2.54]	32 (6.8%)	–0.261	0.948
	net charge	{–2 .. 2}	32 (6.8%)	0.0566	0.712
	hydrophobicity	[–3.37, 3.37]	32 (6.8%)	–0.181	1.16
	Van der Waals volume	[–8.08, 8.08]	32 (6.8%)	–0.185	2.54
	polarizability	[–0.409, 0.409]	32 (6.8%)	–0.0112	0.122
	pK-COOH	[–1.2, 1.2]	32 (6.8%)	–0.245	0.721
	pK-NH2	[–2.29, 2.29]	32 (6.8%)	–0.526	2.32
	hydration	[–5.2, 5.2]	32 (6.8%)	–0.266	1.68
	molecular weight	[–129.17, 129.17]	32 (6.8%)	–7.41	49.4
	optical rotation	[–100.8, 100.8]	32 (6.8%)	5.11	31.3
	secondary structure	{–2 .. 2}	32 (6.8%)	–0.219	1.13
	free energy solution	[–7.15, 7.15]	32 (6.8%)	–0.284	2.08
	# hydrogen bonds	{–4 .. 4}	32 (6.8%)	0.299	1.78
	residue volume	[–169.7, 169.7]	32 (6.8%)	–6.06	63.8
	transfer free energy	[–2.7, 2.7]	32 (6.8%)	–0.203	0.929
	side chain interaction	[–5.64, 5.64]	32 (6.8%)	–0.622	2.36
	# vertices	{–10 .. 10}	32 (6.8%)	–0.233	3.11
	# edges	{–12 .. 12}	32 (6.8%)	–0.258	3.36
	eccentricity	[–11.1, 11.1]	32 (6.8%)	–0.221	3.52
	diameter	[–14, 14]	32 (6.8%)	–0.0475	5.02
	atomic number	[–61, 61]	32 (6.8%)	–2.64	18
*nucleotide-based*	SIFT	[0, 1]	42 (8.9%)	0.154	0.311
	VEST	[0, 1]	129 (27.2%)	0.671	0.291
	PROVEAN	(−∞, +∞)	178 (37.6%)	–3.39	2.68
	FATHMM	[0, 1]	67 (14.1%)	0.696	0.38
	CADD	(−∞, +∞)	42 (8.9%)	3.17	2.18

Ranges for integer feature values are denoted by “#” and listed with a “..” separator between minimum and maximum allowed values, and ranges for float feature values are listed with a “,” separator instead. Percent missing values are calculated out of all 474 prioritized variants. Amino acid-based features are computed as the change in values between reference and alternate alleles; see Methods for further details.

**Table 3:** Performance in distinguishing strongly (positive) from weakly (negative) pathogenic variants in real-world validation set.

Method	Accuracy	Precision	Recall	F-score	AUROC	# Missing
*VarPPUD*	**0.793 (0.031)**	**0.775 (0.034)**	0.829 (0.045)	**0.800 (0.030)**	**0.795 (0.039)**	0 (0.0)
*VarPPUD (no imputation)*	0.779 (0.003)	0.753 (0.039)	0.823 (0.058)	0.786 (0.035)	0.779 (0.037)	0 (0.0)
*PROVEAN*	0.677 (0.081)	0.567 (0.125)	0.802 (0.132)	0.661 (0.120)	0.697 (0.114)	11 (4.9)
*VEST*	0.641 (0.054)	0.617 (0.077)	0.835 (0.076)	0.704 (0.050)	0.641 (0.038)	10 (3.5)
*CADD*	0.637 (0.066)	0.571 (0.081)	0.874 (0.052)	0.687 (0.066)	0.654 (0.059)	2 (1.6)
*Mutation Assessor*	0.614 (0.048)	0.553 (0.095)	0.776 (0.076)	0.642 (0.069)	0.615 (0.071)	18 (3.9)
*SIFT*	0.613 (0.080)	0.502 (0.124)	0.887 (0.064)	0.636 (0.114)	0.657 (0.044)	11 (4.5)
*FATHMM*	0.585 (0.074)	0.514 (0.081)	0.845 (0.104)	0.637 (0.085)	0.609 (0.084)	9 (1.7)
*PolyPhen-2 (HumVar)*	0.574 (0.103)	0.517 (0.108)	0.831 (0.105)	0.632 (0.096)	0.597 (0.105)	22 (3.1)
*Mutation Taster*	0.566 (0.098)	0.517 (0.083)	**0.898 (0.073)**	0.655 (0.081)	0.591 (0.082)	5 (3.7)
*PolyPhen-2 (HumDiv)*	0.554 (0.103)	0.505 (0.100)	0.882 (0.093)	0.636 (0.087)	0.586 (0.093)	22 (3.1)

Performance comparison between two versions of VarPPUD (i.e., with and without feature value imputation) and previous methods for distinguishing strongly from weakly pathogenic variants in real-world patients from the UDN. The number of “missing” variants indicates the number of variants in the validation test that the given method could not generate a prediction for.

### Generating synthetic testing data

We utilized the generative adversarial network-based method CTGAN to generate additional synthetic variants from the unimputed feature vectors of UDN prioritized variants [32]. We specified that all feature values for synthesized data must be within their appropriate ranges and of the correct data type (i.e., integer or float, Table 2) with reasonable constraint design due to the correlation between features. For example, the number of associated rare diseases cannot be greater than the number of total associated diseases. Just like the real-world data they were based on, the resulting 10,000 synthetic variants had some missing feature values, so we randomly selected 250 likely pathogenic and 250 unlikely pathogenic synthetic variants with no missing feature values. We then randomly divided these variants into five groups with 50 likely pathogenic and 50 unlikely pathogenic synthetic variants each, and evaluated VarPPUD’s predictive performance on each of these five synthetic test sets.

### Temporally held-out candidate variants in UDN patients

At the time of manuscript revision, we newly had access to candidate variant information for patients enrolled in the UDN after 2022. Across all patients enrolled from 2022 through 2024, we selected “causative” or “candidate” variants that did not overlap with any prioritized variants used in the training of our model (i.e., found in patients enrolled through 2021). We assigned feature values as before, noting that we use the original per-gene feature values from 2021 (e.g., number of total associated diseases) rather than updated values. Missing feature values were imputed as previously described. This resulted in a set of 95 new variants, 70 of which were “likely pathogenic” and the remaining 25 of which were “unlikely pathogenic”.

### Testing alternate pathogenicity predictors

We compared VarPPUD with nine related variant pathogenicity predictors, five of which were used as input features in our model (i.e., SIFT, VEST4, PROVEAN, FATHMM and CADD [16,17,20,21,23,24]). We also retrieved variant pathogenicity predictions from PolyPhen-2 (HumDiv and HumVar) [14] (http://genetics.bwh.harvard.edu/pph2/bgi.shtml), Mutation Taster (https://www.mutationtaster.org/) [18,19] and Mutation Assessor (http://mutationassessor.org/r3/) [15]. We considered likely and unlikely pathogenic variants as positive and negative examples, respectively, to compute predictive accuracy, precision, recall, F1 score, and area under the receiver operating characteristic curve (AUROC) for each method on the held-out validation set. We also evaluated our model through the area under the precision–recall curve (AUPRC) for external validation with the synthetic data set.

### Measuring feature contribution

We measure how each of our 38 input features contributes toward prediction on our real-world validation set in two ways. First, we randomly shuffled the values for each feature in turn and measured how much overall accuracy on the validation set dropped using the permutation_importance function from Python’s Scikit-learn package [47]. Then, we utilized the Shapley Additive exPlanations (SHAP) package to interpret the contribution of each input feature value (SHAP values) for each individual prediction by the model [35,50]. SHAP values estimate the marginal contribution of each feature by comparing the prediction with and without that feature across many perturbations. Positive SHAP values indicate that a feature contributed toward predicting the variant as more likely pathogenic, whereas negative SHAP values suggest that a feature contributed toward predicting the variant as unlikely pathogenic. Again, these values are calculated on a per-variant basis, enabling us to visualize feature importance for individual predictions. To summarize the overall (rather than per-prediction) contribution of each feature, we computed the mean of the absolute SHAP values for each feature across all variants in the validation set. This yielded an overall ranking of features based on their average contribution to prediction, regardless of direction (i.e., likely vs. unlikely pathogenic). Finally, we computed SHAP interaction values, capturing how pairs of feature values contribute to prediction. These interaction values help identify feature pairs that may work synergistically or antagonistically in the model’s decision-making process.

### Incorporating patient demographic features

Each of the starting 222 likely pathogenic and 252 unlikely pathogenic variants from real-world UDN patients are associated with additional patient information, including age at first symptom onset, “current” age at evaluation, self-identified gender, self-identified ethnicity (Hispanic/Latino or otherwise), and self-identified race. For each of these features, we retrained VarPPUD ten times using the original 38 features and the new demographic feature. Each retraining used a different random seed to split the starting variants into training and validation sets in a 9:1 ratio. Changes to predictive performance was measured as the change in area under the receiver-operator curve compared to the version of VarPPUD with no demographic features.

## Results

### Patient demographics

We trained a new variant pathogenicity predictor, VarPPUD, on 474 unique variants impacting 413 genes that were originally prioritized through extensive manual and computational efforts in 474 patients enrolled in the Undiagnosed Diseases Network (UDN) [10]. These patients’ symptoms began at age 6.1 on average, and they endured multiple failed diagnostic attempts for nearly 9 years on average before being accepted into and evaluated by the UDN (Table 1). As expected, the genetic variants eventually prioritized in their cases are extremely unique and often had never been previously identified as disease-causing [9]. The candidate and causative genetic variants from these patients were designated by clinical teams as likely pathogenic (n=222) or otherwise (called unlikely pathogenic [n=252] herein); VarPPUD was trained to discriminate between these two classes (see Methods for details). Our cohort of patients included an equivalent number of female and male patients (232 [48.9%] vs. 242 [51.1%]) with a majority classified as white (356 [76.1%]). Patients’ primary symptoms in both the likely and unlikely pathogenic sets were mostly neurologic (118 [53.9%] vs. 107 [43.1%], respectively) or musculoskeletal (38 [17.4%] vs. 34 [13.7%], respectively). Variants in patients with primarily rheumatologic (n=10), oncologic (n=2) or craniofacial (n=2) symptoms were exclusively in the unlikely pathogenic class. Overall, patients’ primary symptom categories between the two variant classes were evenly distributed, with an average ratio of 1:1.2 between the likely and unlikely pathogenic variant classes across all primary symptom categories.

### VarPPUD’s predictive ability on real-world and synthetic variants

**Fig 2:**
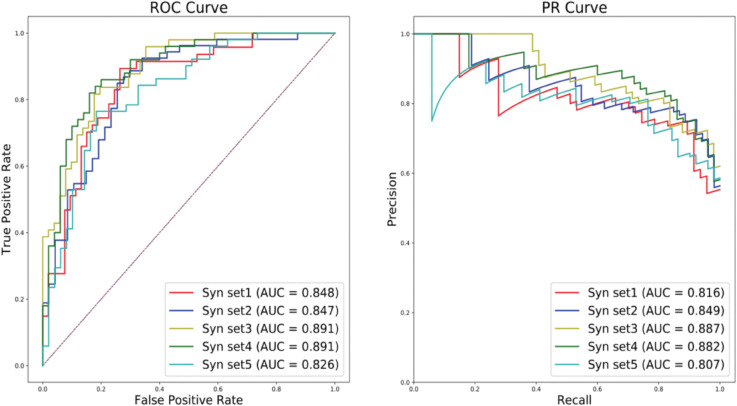
VarPPUD’s predictive performance on five GAN-synthesized variant datasets. Performance of the proposed model at identifying strong pathogenic variants on five different GAN-based synthetic testing sets evaluated by Receiver Operating Characteristic (ROC) and Precision-Recall (PR) curves.

We held out 10% of the real-world prioritized variants as a validation set, and used the remaining 90% of the data to train and test VarPPUD. Our model achieved an overall classification accuracy of 0.793 and a precision in detecting likely pathogenic variants of 0.775 on the validation set (Table 3). We also evaluated VarPPUD’s predictive performance on a real-world validation set of candidate variants from UDN patients enrolled after the development of the original model, from 2022–2024. We filtered variants as before (Fig 1A) and ensured that no variants in this validation set overlapped with any variants used in training or testing of the original model. VarPPUD achieved an overall classification accuracy of 0.779 and a precision in detecting likely pathogenic variants of 0.765 on this real-world validation set. Finally, due to the small number of variants in our validation set, we also generated five sets of synthetic variants using a GAN-based technique (see Methods for details); each synthetic test set contained 50 likely and 50 unlikely pathogenic variants. VarPPUD achieved an area under the receiver-operator characteristic curve (AUROC) of 0.826 to 0.891 (0.860 on average) across these synthetic test sets, and an area under the precision-recall curve (AUPRC) of 0.807 to 0.887 (0.848 on average) with respect to likely pathogenic variants (Fig 2). These results suggest that VarPPUD can stably discriminate between likely pathogenic and unlikely-to-be-confirmed pathogenic variants in both real-world and synthetic datasets.

### Comparison to related pathogenicity predictors

VarPPUD is trained on a unique set of variants that were all initially prioritized due to their potential to cause disease; discriminating between likely and unlikely pathogenic variants within this set is a goal that existing pathogenicity predictors were not specifically designed nor optimized for. Moreover, some existing pathogenicity predictors only generate predictions for specific variant types (e.g., PolyPhen-2, SIFT), and therefore could not generate predictions for two (CADD) to 22 (PolyPhen-2) variants in our validation set. Nevertheless, we sought to compare VarPPUD’s ability to distinguish likely from unlikely pathogenic variants across all variant types—including large protein-coding indels, nonsense and synonymous variants—to the ability of nine related pathogenicity predictors to perform this same task. Estimating the pathogenicity of all variant types is especially desirable in still-undiagnosed cases where known, disease-causing, protein-altering SNV/indel variants were not uncovered. To this end, we computed pathogenicity or deleteriousness predictions for all variants in our validation set, where possible, using SIFT [17], VEST4 [21], PROVEAN [16], FATHMM [20], CADD [24], Mutation Taster [19], Mutation Assessor [15], and two versions of PolyPhen-2 (i.e., disease variants are differentiated from variants at divergent sites across close mammalian homologs [HumDiv] or from common human polymorphisms unassociated with disease [HumVar]) [14]. We found that VarPPUD achieved higher overall prediction accuracy, higher precision in identifying likely pathogenic variants, a higher F-score, and a higher AUROC than all other methods on the held-out validation set of real-world prioritized variants (Table 3). However, all but two methods (PROVEAN and Mutation Assessor) achieved higher recall of likely pathogenic variants than VarPPUD, with Mutation Taster achieving the highest recall across all methods. As previously mentioned, these alternate predictors were trained to distinguish pathogenic from benign (rather than from unlikely pathogenic) variants across the whole genome, rather than within a prioritized variant list, and therefore would predict nearly all the variants in our validation set to be likely pathogenic. As such, it is expected that they would have higher recall of likely pathogenic variants within this set. Nevertheless, VarPPUD achieved a comparably high recall of 0.829 on our validation set. Moreover, we found that although the imputation of missing feature values improved VarPPUD’s predictive performance, even the version of VarPPUD without feature value imputation better identified likely pathogenic variants relative to other existing tools on nearly all evaluation metrics. These results demonstrate the relatively superior ability of VarPPUD to distinguish likely from unlikely pathogenic variants within a set of prioritized, candidate disease-causing variants.

### Importance and contribution of VarPPUD features

**Fig 3:**
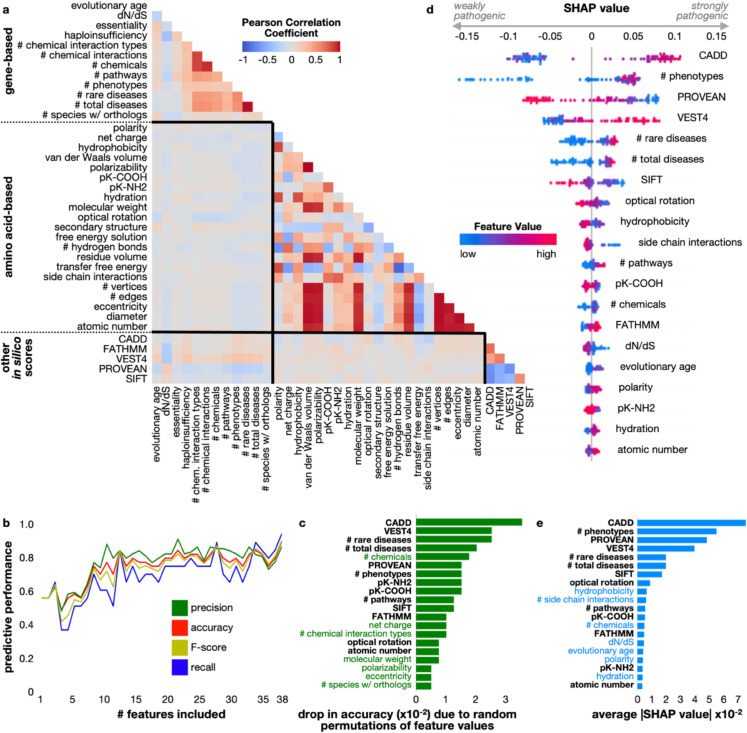
Feature correlation and contribution toward prediction. A: Correlations between feature values were computed for all variants in the real-world training dataset using Pearson’s Correlation Coefficient. Feature names are grouped by category: gene-based, amino acid-based, and nucleotide-based (*in silico* deleteriousness predictions) and separated by black lines in the heatplot. B: Features were eliminated in the same order as Table 2 and the model was retrained with five-fold cross validation on the training set; accuracy (red), precision (green), recall (blue), and F-score (yellow) were computed from performance on the held-out test set. C: Feature importance measured as the drop in accuracy on the test set when values for that feature were randomly permuted in the training set. Feature names highlighted in green indicate importance by this measure and not by the measure in (E). D: SHAP values for each feature in each individual prediction on the real-world test set. Negative SHAP values indicate contribution toward predicting a variant to be weakly pathogenic, whereas positive SHAP values indicate a feature’s contribution toward predicting a variant to be strongly pathogenic. Points are colored by their linearly normalized (between 0 and 1) feature value, organized from low (blue) to high (red). E: Feature importance measured as the average of the absolute values of the SHAP values computed for all variants in the test set; feature names highlighted in blue indicate importance by this measure and not by the measure in (D).

We next wanted to evaluate the importance of each of VarPPUD’s features. VarPPUD incorporates 12 gene-level features (e.g., number of associated diseases or pathways, evolutionary age, essentiality and haploinsufficiency), 21 features capturing putative physicochemical changes due to amino acid substitutions, and five deleteriousness scores from existing *in silico* methods (see Methods for details). Although some amino acid-based features were highly correlated with each other as expected (e.g., polarity decreases as number of hydrogen bonds increases, Pearson Correlation Coefficient [PCC] > 0.9, Fig 3A), most features showed low correlations with each other, particularly between features from different categories (-0.1 < PCC < 0.1). The five *in silico* deleteriousness features tended to be most highly correlated with each other, with CADD, FATHMM, and VEST4 showing the lowest relative correlations among each other and SIFT and PROVEAN showing the highest relative correlations. Overall, most of VarPPUD’s input features are uncorrelated and thus have the potential to contribute complementary information relevant for variant pathogenicity prediction.

We first investigated how individual features contribute to VarPPUD’s overall discriminatory power. Note that because we impute missing feature values for all variants, VarPPUD’s discriminatory performance would not be due to any consistent differences in feature value availability between the two variant classes (Table 3). To this end, we trained ablated versions of VarPPUD using subsets of input features and evaluated the resulting models’ performance using precision, accuracy, F-score and recall of likely pathogenic variants. Specifically, we increasingly included each feature one by one, starting with gene-based features, then also including amino acid-based features, and finally including nucleotide variant-based features in the same order as listed in Table 2. Each new predictor was evaluated using synthetic test sets as before. As expected, we found that the best performance was achieved by the version of VarPPUD that utilized all 38 features (accuracy of 0.813, Fig 3B). Intriguingly, although the inclusion of more features resulted in a general trend of better performance, we found that iteratively including new features individually did not always result in improved performance. For instance, including the total number of gene–chemical interactions for a gene as a feature resulted in a worse-performing predictor than including only the total number of phenotypes associated with the gene, the number of pathways the gene is a part of, and the number of unique chemicals the gene interacts with. We hypothesized that the interdependency of some features may require pairs or groups of features to be present to be exploited for a discriminatory advantage by our random forest model.

Since the version of VarPPUD that utilized all 38 features had the best performance, we next excluded each feature one-by-one to better understand the relative importance of each feature based on the overall drop in predictive performance. Specifically, we permuted the values for each feature in turn across all variants in the synthetic test sets and measured VarPPUD’s predictive accuracy (Fig 3C). We found that values for nucleotide-level CADD and VEST4 features and values for the number of total and rare diseases associated with the gene were most important for accurately determining whether a prioritized variant was likely or unlikely to be pathogenic.

### Asymmetric influence of features to model prediction

Certain values of some features may strongly implicate a variant as likely pathogenic, but certain values for an entirely different set of features may strongly indicate that a variant is unlikely pathogenic. To assess these potential differences and evaluate how and whether specific feature values impact the directionality of VarPPUD’s predictions, we next generated and analyzed Shapley Additive exPlanations (SHAP) values (Fig 3D). Briefly, for each individual prediction, a negative SHAP value for a specific feature suggests that the value of that feature contributed to a prediction of unlikely pathogenic, whereas a positive SHAP value for a specific feature suggests that that feature’s value contributed to a prediction of likely pathogenic. By overlaying SHAP values with feature values, we can see whether high or low feature values contribute to a likely or unlikely pathogenic prediction. As expected, we found that high and low CADD scores respectively corresponded to likely and unlikely pathogenic predictions. In contrast however, we found that although a small number of associated gene phenotypes often strongly contributed to a prediction of unlikely pathogenic, many associated gene phenotypes did not necessarily indicate a prediction of likely pathogenic. The number of rare and total diseases had a similarly asymmetric impact; small numbers of associated diseases with a gene resulted in a strong prediction of unlikely pathogenic, but a relatively large number of total and rare associated diseases did not indicate a prediction of likely pathogenic. It is well established that larger numbers of annotated phenotypes and associated diseases with a gene contribute to the likelihood of variants within that gene being pathogenic overall. However, once a set of candidate variants across the genome has been prioritized for a particular patient, these features are no longer as important for selecting the exact disease-causing variant from the other candidates. Our analysis of VarPPUD’s feature importance in this manner illustrates how distinguishing likely from unlikely pathogenic variants within a set of prioritized, candidate variants is an inherently different task from identifying pathogenic from benign variants genome-wide, and some unique features with counterintuitive values may be leveraged for this specific task. We did not find striking examples of asymmetric contributions to prediction when looking at products of feature values (S1 Fig).

Finally, we assessed the mean absolute values of SHAP values per feature across all predictions as another measure of overall feature importance. We found that ranking features by importance using this metric differed from ranking features by overall accuracy drop when feature values were randomized (Fig 3E). Changes to amino acid hydrophobicity and the number of side chain interactions, for instance, had relatively high average SHAP values, yet these features did not appear to result in a large performance drop when their values were randomized. Indeed, many of the features uniquely ranked by the average SHAP value procedure appear to correspond to features where certain values can be informative for predicting one variant class, but less informative for the other variant class. Although several individual features and pairwise combinations of features contribute strongly to prediction, it is possible that some features utilized by VarPPUD were not particularly useful in distinguishing between the pathogenicity classes described here. In general, physicochemical features of amino acid substitutions influenced pathogenicity prediction to a lesser extent than gene- and nucleotide-level features.

### Patient demographics are not predictive of pathogenicity

**Fig 4:**
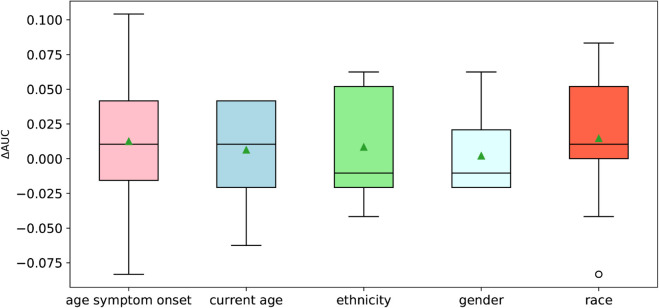
Change in performance when including patient demographics as features. We retrained VarPPUD five times, including one new demographic feature each time. We compute the area under the ROC curve (ΔAUROC) ten times for each retrained model. Horizontal lines within each boxplot indicate the median value, green triangles indicate the mean value, and white circles indicate outliers.

We explored whether VarPPUD’s ability to discern likely from unlikely pathogenic variants could be improved by incorporating patient demographic information alongside the gene-, amino acid- and nucleotide-level features used (Fig 4). Although the age at first symptom onset, current age, and patient-recorded race showed promise in marginally increasing predictive capability, these values did not consistently improve model performance. Incorporating the age at first symptom onset resulted in the highest fluctuation in performance, and incorporating ethnicity and patient-recorded gender reduced overall predictive performance. Although we did not pursue further incorporation of patient-level features, it is possible that a more systematic and extensive effort to this end beyond the scope of this paper could be fruitful.

### Pathogenicity predictions for variants in the same genes

Candidate variants impacting the same gene across unrelated patients showing similar symptoms can sometimes be upgraded from uncertain significance to definitively causing disease. For example, three different variants in the gene CACNA1A (c.1360C>T, c.4055G>T, c.5018G>C) found in unrelated UDN patients with primarily neurological symptoms were eventually confirmed as disease-causing. Similarly, three variants in MECP2 gene (c.538C>T, c.316C>T, c.352C>T) identified in unrelated UDN patients with symptoms akin to Rett syndrome and severe encephalopathy were reclassified as pathogenic by the overseeing clinical teams. VarPPUD correctly predicted these variants as likely pathogenic. However, it is important to note that not all candidate variants in the same gene are necessarily relevant to disease in every patient. For instance, two variants in the NOD2 gene (c.2104C>T and c.2798+158C>T) were initially categorized as uncertain despite being found in two unrelated UDN patients with rheumatosis, an immune-related condition impacting the joints, muscles and ligaments. Another UDN patient with primarily immunological symptoms had a different NOD2 variant, c.1292C>T. VarPPUD predicted all three NOD2 variants as pathogenic. These variants have since been reclassified as likely pathogenic or pathogenic, showcasing VarPPUD’s potential as a supportive tool in reevaluating the pathogenicity of variants of uncertain significance.

## Discussion

Identifying a set of putative disease-causing variants in patients with undiagnosed genetic conditions is a challenging, multi-step process [10]. Variant prioritization often involves the genome-wide application of *in silico* pathogenicity and deleteriousness predictors to exclude common, likely benign variants from downstream consideration. Even after filtering out likely benign variants and restricting to variants in genes that seem relevant to a patient’s phenotypic presentation, variant prioritization pipelines can still return dozens of variants of uncertain significance [51,52]. In practice, these candidate variants are manually investigated by clinical experts and confirmed or rejected as causal through extensive functional analyses and variant matchmaking services [53]. In our work, we accessed a unique dataset of uncertain candidate variants originally prioritized through genomic analysis of patients enrolled in the Undiagnosed Diseases Network (UDN) and eventually reclassified as benign or pathogenic. We trained a binary classifier, VarPPUD, to categorize whether any strong candidate variants were likely to be confirmed as disease-causing or not based on nucleotide-, amino acid-, and gene-level properties. VarPPUD achieved superior performance on this classification task on both real and GAN-generated synthetic data compared to existing methods that were developed to distinguish deleterious from otherwise benign variants. Our results show that the task of selecting the single disease-causing variant from a set of already compelling variants is different from the first-pass, genome-wide prioritization task for which most pathogenicity predictors have been developed. Our model analysis revealed that some features utilized by VarPPUD to gain discriminatory power—such as the number of rare or total disorders associated with the gene—are used in ways that may appear counterintuitive when compared to traditional predictors trained to distinguish pathogenic from benign variants genome-wide. VEST, for instance, is another random forest model trained on 45,000 disease mutations from the Human Gene Mutation Database and 45,000 common, likely neutral missense variants from the Exome Sequencing Project [21,26,54]. Despite its substantially larger training set size, VEST was less suited to distinguish likely from unlikely pathogenic variants relative to VarPPUD when applied to strong candidate variants from UDN patients.

The diagnosis rate for new patients with suspected but elusive genetic conditions hovers around 30% [9]. Once patients have had inconclusive clinical sequencing, reanalysis or reinterpretation of their sequencing data is rarely automated, and even patients with strong candidate variants can remain in an undiagnosed limbo for many years. As the number of sequenced rare disease patients continues to grow, automated reanalysis and reinterpretation of variant findings will be essential for improving diagnostic rates. We propose that VarPPUD can be applied as a second-line pathogenicity predictor to select for disease-causing variants from among the sets of prioritized candidate variants in patients where progress has otherwise stalled.

We acknowledge several limitations of the work we present here. Although VarPPUD successfully makes predictions on indel variants whereas other missense-only predictors such as PolyPhen2 or SIFT fall short, there are two primary shortcomings to this functionality. First, the amino acid feature values we compute for indels may not be as biologically interpretable as those computed for SNVs. For instance, inserting an amino acid into a protein alpha-helix structure may more severely disrupt protein secondary structure than would be captured by our scoring technique. Second, despite VarPPUD’s functionality in predicting pathogenicity of indels, the training data we used was primarily composed of missense variants. Indeed, variants prioritized in short-read clinical genome sequencing data prior to 2021 were often SNVs, as indel-calling accuracy was relatively worse [55]. Moreover, missense SNVs were more difficult to interpret, and therefore more likely to be represented in the UDN dataset, as compared to truncating mutations, because missense impact on protein structure and function is less clear [56]. Nevertheless, we find that being able to score indel variants in this manner leads to better overall performance and enables VarPPUD to score a more realistic set of strong candidate variants that would be prioritized for a rare disease patient. Moreover, although VarPPUD makes predictions for variant types beyond SNVs, it does not score certain disease-causing genetic aberrations such as large structural rearrangements, gene duplication events, or expansions of repeat regions.

We impute feature values such as per-gene haploinsufficiency or gene essentiality for variants where those values are missing. Although imputed values are logically constrained and their inclusion improves the overall predictive performance of our model, imputed values may be incorrect and generally detract from our ability to meaningfully interpret specific predictions. Even if independent empirical data could be used to predict missing feature values instead of the MICE approach employed here, resulting values may still be misleading or clinically irrelevant [57,58]. Independently confirming the accuracy of imputed feature values is beyond the scope of this work. Some of the most informative features used by VarPPUD are nucleotide-level deleteriousness scores produced by other variant effect predictors including CADD and PROVEAN. Meaningfully interpreting specific predictions is further hindered in cases where these ensemble scoring metrics are used to distinguish likely from unlikely pathogenic variants.

There are still some variants misclassified by VarPPUD. The c.2121delT variant in the IL6ST gene, for example, was miscategorized as a likely pathogenic variant by VarPPUD. This indel variant (deletion of a single thymine nucleotide) results in a frameshift effect, which tends to be damaging. Nevertheless, this particular variant was not etiological for this UDN patient, demonstrating the difficulty in reliably identifying truly disease causative variants from strong candidate variants by a purely computational approach.

Finally, we trained VarPPUD on a relatively small set of 474 candidate variants across 413 unique genes. We show high discriminatory performance on a held-out validation set of UDN data, on an independent, temporally distinct set of UDN data, and on GAN-generated synthetic variant datasets based on UDN data. We recognize that candidate variants prioritized by other, non-UDN frameworks might be sufficiently or critically different from the UDN-based training data used here. We also note that, per our patient inclusion criteria (Fig 1A), only one prioritized variant had been manually entered per patient, and that these prioritized variants were well balanced between likely versus unlikely pathogenic. It is feasible that end-users of our tool might start with multiple computationally-prioritized variants per case, with only one being truly diagnostic. It is also possible that multiple variants are disease causative for a rare, genetic condition. The training data used during model development did not represent either of these cases, and therefore VarPPUD’s generalizability to these cases remains untested. However, the UDN is composed of multiple, independent clinical research sites employing distinct, in-house prioritization pipelines, so the variants prioritized network-wide used here are likely to be generally representative of the types of variants that would be uncovered by state-of-the-art prioritization pipelines.

## Supporting information

S1 Fig SHAP interaction scores for the most important 20 features that predict variant pathogenicity in undiagnosed patients. SHAP values for each pair of features in each individual prediction on the real-world test set. Negative SHAP values indicate contribution toward predicting a variant to be weakly pathogenic, whereas positive SHAP values indicate a feature’s contribution toward predicting a variant to be strongly pathogenic. Points are colored by their linearly normalized (between 0 and 1) feature value, organized from low (blue) to high (red).
(TIFF)

S1 Table Description of physicochemical and biochemical properties of amino acids derived from AAindex. (DOCX)

S1 Text Undiagnosed Diseases Network Consortium Members, Version 8.13.25. Alphabetized roster of Undiagnosed Diseases Network members as of August 13, 2025, with UDN-specific affiliations.
(DOCX)
