# Supplementary figures and images for: VarPPUD: Pinpointing diagnostic variants from sets of prioritized, strong candidate variants
Source: PLoS Comput Biol. 2025 Sep 22;21(9):e1013414. doi: 10.1371/journal.pcbi.1013414 (PMC12468739; doi:10.1371/journal.pcbi.1013414)

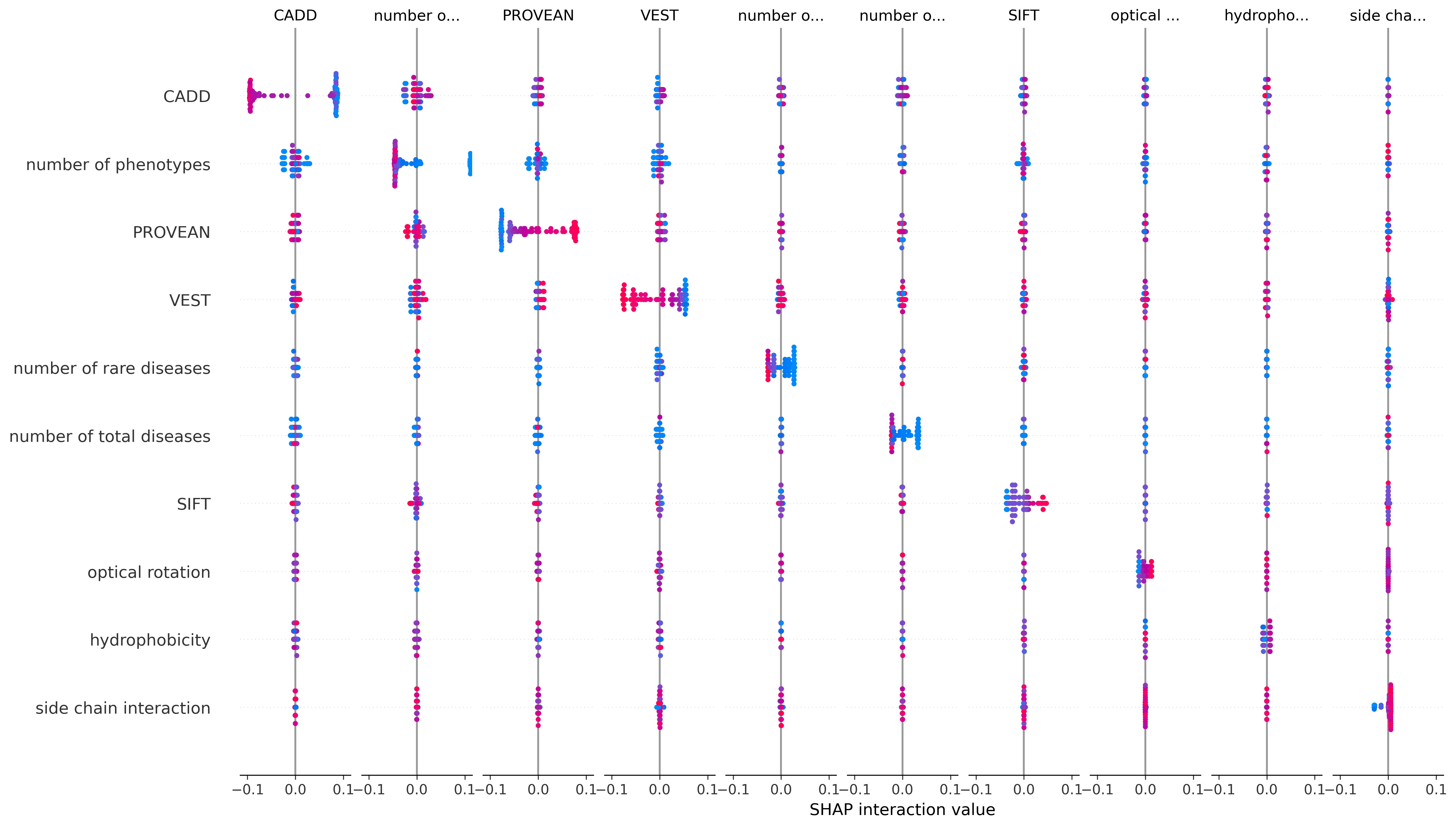

Supplement: S1 Fig — SHAP values for each pair of features in each individual prediction on the real-world test set. Negative SHAP values indicate contribution toward predicting a variant to be weakly pathogenic, whereas positive SHAP values indicate a feature’s contribution toward predicting a variant to be strongly pathogenic. Points are colored by their linearly normalized (between 0 and 1) feature value, organized from low (blue) to high (red). (TIFF) [file pcbi.1013414.s001.tiff]
