# Supplementary material for: VarPPUD: Pinpointing diagnostic variants from sets of prioritized, strong candidate variants
Source: PLoS Comput Biol. 2025 Sep 22;21(9):e1013414. doi: 10.1371/journal.pcbi.1013414 (PMC12468739; doi:10.1371/journal.pcbi.1013414)
Supplement: S1 Table — (DOCX) [file pcbi.1013414.s002.docx]

**S1 Table. Description of physicochemical and biochemical properties of amino acids derived from AAindex.**

| Entry | Property | Literature | Reference |
| --- | --- | --- | --- |
| RADA880108 | Polarity | Side-chain distribution coefficients between the vapor phase, cyclohexane, 1-octanol, and neutral aqueous solution | (1) |
| KLEP840101 | Net charge | Prediction of protein function from sequence properties: Discriminant analysis of a data base | (2) |
| ﻿CIDH920103 | Hydrophobicity | Hydrophobicity and structural classes in proteins | (3) |
| ﻿FAUJ880103 | Normalized van der Waals volume | Amino acid side chain parameters for correlation studies in biology and pharmacology | (4) |
| ﻿CHAM820101 | ﻿Polarizability | The structural dependence of amino acid hydrophobicity parameters | (5) |
| ﻿JOND750102 | ﻿pK-COOH | Amino acid properties and side-chain orientation in proteins: A cross correlation approach | (6) |
| FASG760104 | pK-NH2 | Handbook of Biochemistry and Molecular Biology | (7) |
| ﻿ROBB790101 | Hydration free energy | Refined models for computer simulation of protein folding: Applications to the study of conserved secondary structure and flexible hinge points during the folding of pancreatic trypsin inhibitor | (8) |
| ﻿FASG760101 | Molecular weight | Handbook of Biochemistry and Molecular Biology | (8) |
| ﻿FASG760103 | Optical rotation | Handbook of Biochemistry and Molecular Biology | (8) |
| ﻿LEVJ860101 | ﻿Secondary structure | An algorithm for secondary structure determination in proteins based on sequence similarity | (9) |
| CHAM820102 | ﻿Free energy of solution in water | The structural dependence of amino acid hydrophobicity parameters | (5) |
| FAUJ880109 | Number of hydrogen bond donors | Amino acid side chain parameters for correlation studies in biology and pharmacology | (4) |
| ﻿PONJ960101 | ﻿Average volumes of residues | Deviations from standard atomic volumes as a quality measure for protein crystal structures | (10) |
| ﻿JANJ790102 | Transfer free energy | Surface and inside volumes in globular proteins | (11) |
| ﻿WARP780101 | Average interactions per side chain atom | A survey of amino acid side-chain interactions in 21 proteins | (12) |
| ﻿KARS160101 | Number of vertices | A graph-theoretic model of single point mutations in the cystic fibrosis transmembrane conductance regulator | (13) |
| ﻿KARS160102 | ﻿Number of edges | A graph-theoretic model of single point mutations in the cystic fibrosis transmembrane conductance regulator | (13) |
| ﻿KARS160105 | Eccentricity | A graph-theoretic model of single point mutations in the cystic fibrosis transmembrane conductance regulator | (13) |
| ﻿KARS160107 | Diameter | A graph-theoretic model of single point mutations in the cystic fibrosis transmembrane conductance regulator | (13) |
| ﻿KARS160117 | Total weighted atomic number | A graph-theoretic model of single point mutations in the cystic fibrosis transmembrane conductance regulator | (13) |

**References**

1. Radzicka A, Wolfenden R. Comparing the polarities of the amino acids: side-chain distribution coefficients between the vapor phase, cyclohexane, 1-octanol, and neutral aqueous solution[J]. Biochemistry, 1988, 27(5): 1664-1670.
2. Klein P, Kanehisa M, DeLisi C. Prediction of protein function from sequence properties: Discriminant analysis of a data base[J]. Biochimica et Biophysica Acta (BBA)-Protein Structure and Molecular Enzymology, 1984, 787(3): 221-226.
3. Cid H, Bunster M, Canales M, et al. Hydrophobicity and structural classes in proteins[J]. Protein Engineering, Design and Selection, 1992, 5(5): 373-375.
4. FAUCHÈRE J L U C, Charton M, Kier L B, et al. Amino acid side chain parameters for correlation studies in biology and pharmacology[J]. International journal of peptide and protein research, 1988, 32(4): 269-278.
5. Charton M, Charton B I. The structural dependence of amino acid hydrophobicity parameters[J]. Journal of theoretical biology, 1982, 99(4): 629-644.
6. Jones D D. Amino acid properties and side-chain orientation in proteins: a cross correlation approach[J]. Journal of theoretical biology, 1975, 50(1): 167-183.
7. Fasman G D. Practical handbook of biochemistry and molecular biology[M]. CRC press, 1989.
8. Robson B, Osguthorpe D J. Refined models for computer simulation of protein folding: Applications to the study of conserved secondary structure and flexible hinge points during the folding of pancreatic trypsin inhibitor[J]. Journal of molecular biology, 1979, 132(1): 19-51.
9. Levin J M, Robson B, Garnier J. An algorithm for secondary structure determination in proteins based on sequence similarity[J]. FEBS letters, 1986, 205(2): 303-308.
10. Pontius J, Richelle J, Wodak S J. Deviations from standard atomic volumes as a quality measure for protein crystal structures[J]. Journal of molecular biology, 1996, 264(1): 121-136.
11. Janin J. Surface and inside volumes in globular proteins[J]. Nature, 1979, 277(5696): 491-492.
12. Warme P K, Morgan R S. A survey of amino acid side-chain interactions in 21 proteins[J]. Journal of molecular biology, 1978, 118(3): 289-304.
13. Kakraba S, Knisley D. A graph-theoretic model of single point mutations in the cystic fibrosis transmembrane conductance regulator[J]. Journal of Advances in Biotechnology, 2016, 6(1): 780-786.
