# Supplementary material for: VarPPUD: Pinpointing diagnostic variants from sets of prioritized, strong candidate variants
Source: PLoS Comput Biol. 2025 Sep 22;21(9):e1013414. doi: 10.1371/journal.pcbi.1013414 (PMC12468739; doi:10.1371/journal.pcbi.1013414)
Supplement: S1 Text — Alphabetized roster of Undiagnosed Diseases Network members as of August 13, 2025, with UDN-specific affiliations. (DOCX) [file pcbi.1013414.s003.docx]

**S1 Acknowledgements. Undiagnosed Diseases Network Consortium Members, Version** **8.13.25**

Jose Abdenur^3^, Maria T. Acosta^4^, David R. Adams^4,5^, Ben Afzali^4,5^, Eric Allenspach^6^, Raquel L. Alvarez^7^, Justin Alvey^8^, Ashley Andrews^8^, Beatriz Anguiano^7^, Euan A. Ashley^9^, Sanaz Attaripour^3^, Paul Auwaerter^10^, Suha Bachir^7^, Carlos A. Bacino^11^, Guney Bademci^12^, Ashok Balasubramanyam^11^, Dustin Baldridge^13^, Erin E. Baldwin^8^, Allen Bale^14^, Elsa Balton^6^, Manisha Balwani^15^, Michael Bamshad^6^, Mafalda Barbosa^15^, Deborah Barbouth^12^, Rebekah Barrick^3^, Donald Basel^16^, Lisa Bastarache^17^, Pinar Bayrak-Toydemir^18^, Taylor Beagle^19^, Alan H. Beggs^20^, Edward Behrens^21^, Megan Bell^19^, Hugo J. Bellen^22^, Zackary Dov Berger^10^, Paul Berger^19^, Jonathan A. Bernstein^7^, Gerard T. Berry^20^, Louise Bier^15^, Stephanie Bivona^12^, Reaford Blackburn^23^, Kirsten Blanco^3^, Lauren Blieden^11^, Elizabeth Blue^6^, Devon Bonner^7^, Brett Bordini^16^, Nicholas Borja^12^, Lorenzo Botto^8^, Steven Boyden^8^, Lauren C. Briere^20^, Elizabeth A. Burke^4,5^, Lindsay C. Burrage^11^, Francisco Bustos^19^, Manish J. Butte^24^, Russell Butterfield^8^, Peter Byers^6^, William E. Byrd^25^, Kaitlin Callaway^23^, John Carey^8^, George Carvalho^24^, Thomas Cassini^17^, Chun-Hung Chan^19^, Richard Chang^3^, Sirisak Chanprasert^6^, Hsiao-Tuan Chao^11^, Elizabeth C. Chao^3^, Ivan Chinn^11^, Gary D. Clark^11^, Terra R. Coakley^7^, Laurel A. Cobban^20^, Joy D. Cogan^17^, Matthew Coggins^20^, F. Sessions Cole^26^, Erin Conboy^27^, Rosario I. Corona^24^, William J. Craigen^11^, Andrew B. Crouse^25^, Vishnu Cuddapah^21^, Charlotte Cunningham-Rundles^15^, Precilla D’Souza^4^, Hongzheng Dai^11^, Nitsuh K. Dargie^6^, Kahlen Darr^28^, Surendra Dasari^28^, Erica Davis^29^, Joie Davis^4,5^, Margaret Delgado^4,5^, Esteban C. Dell’Angelica^24^, Nada Derar^14^, Patricia Dickson^30^, Katrina Dipple^6^, Naghmeh Dorrani^24^, Jessica Douglas^20^, Abdul Elkadri^16^, Sara Emami^7^, Lisa T. Emrick^11^, Christine M. Eng^31^, Cecilia Esteves^32^, Rachel Evard^15^, Kimberly Ezell^17^, Layal F. Abi Farraj^24^, Elizabeth L. Fieg^20^, Paul G. Fisher^7^, Brent L, Fogel^24^, Jiayu Fu^4,5^, William A. Gahl^4,5^, Eric Gamazon^17^, Rebecca Ganetzky^21^, Eric Gayle^15^, Bruce Gelb^15^, Mark Gerstein^14^, Emily Glanton^32^, Ian Glass^6^, Page C. Goddard^7^, Joanna M. Gonzalez^12^, John E. Gorzynski^7^, Brett H. Graham^27^, Andrea Gropman^4^, Ziyuan Guo^33^, Meghan C. Halley^7^, Winston Halstead^14^, Rizwan Hamid^17^, Neil Hanchard^4,5^, Kelly Hassey^21^, Caroline Hendry^14^, Frances High^20^, Fuki M. Hisama^6^, Ingrid A. Holm^20^, Jason Hom^7^, Julie Hoover-Fong^10^, Martha Horike-Pyne^6^, Yan Huang^4,5^, Alden Huang^24^, Monika Weisz Hubshman^11^, Anna Hurst^23^, John A. Phillips III^17^, Wendy Introne^4,5^, Ayuko Iverson^15^, Gail P. Jarvik^6^, Orpa Jean-Marie^4,5^, Lauren Jeffries^14^, Joanna Jen^15^, Tanner D. Jensen^7^, Yong-Hui Jiang^14^, Vaidehi Jobanputra^34^, Oguz Kanca^22^, Yigit Karasozen^24^, Odelya Kaufman^14^, Laura Keehan^7^, Shamika Ketkar^11^, Dana Kiley^30^, Gonench Kilich^21^, Eric Klee^28^, Shilpa Nadimpalli Kobren^1,33^, Isaac S. Kohane^32^, Jennefer N. Kohler^7^, Bruce Korf^23^, Susan Korrick^20^, Mary Koziura^17^, Elijah Kravets^7^, Alyson Krokosky^17^, Runjun Kumar ^6^, Seema R. Lalani^11^, Brendan C. Lanpher^28^, Ian R. Lanza^28^, Kumarie Latchman^12^, Kimberly LeBlanc^32^, Brendan H. Lee^11^, Miranda Leitheiser^19^, Monkol Lek^14^, Kathleen A. Leppig^6^, Pongtawat Lertwilaiwittaya^23^, Mia Levanto^7^, Richard A. Lewis^11^, Rachel Li^19^, Khurram Liaqat^27^, Pengfei Liu^31^, Nicola Longo^8^, Joseph Loscalzo^20^, Richard L. Maas^20^, Ellen F. Macnamara^4^, Calum A. MacRae^20^, Valerie V. Maduro^4^, MayChristine V. Malicdan^4,5^, Tarun K. K. Mamidi^23^, Yuka Manabe^10^, Shrikant Mane^14^, Lili Mantcheva^27^, Rong Mao^18^, Ronit Marom^11^, Julian A. Mart´ınez-Agosto^24^, Gabor Marth^35^, Beth A. Martin^7^, Martin G. Martin^24^, Shruti Marwaha^7^, Taylor Maurer^7^, Christopher Mayhew^33^, Julie McCarrier^16^, Allyn McConkie-Rosell^36^, Ashley McMinn^17^, Patrick McMullen^29^, Erin McRoy^30^, Hector Rodrigo Mendez^7^, Eneida Mendonca^33^, Matthew Might^25^, Mohamad Mikati^36^, Danny E. Miller^6^, Alexander Miller^7^, Ghayda Mirzaa^6^, Breanna Mitchell^28^, Stephen B. Montgomery^7^, Paolo Moretti^8^, Jennifer Morgan^19^, Marie Morimoto^4,5^, Tahseen Mozaffar^3^, Lindsay Mulvihill^28^, John J. Mulvihill^4^, Michael Muriello^16^, Sandesh Nagamani^11^, Mariko Nakano-Okuno^25^, Stanley F. Nelson^24^, Thomas J. Nicholas^8^, Arian Nouraee^29^, Donna Novacic^4^, Devin Oglesbee^28^, Carol Oladele^14^, James P. Orengo^11^, Rebecca Overbury^8^, Kathleen Page^10^, Stephen C. Pak^13^, J. Carl Pallais^20^, Carlos A. Pardo-Villamizar^10^, Neil H. Parker^24^, Alex Paul^30^, L´eShon Peart^12^, Lakshitha Perera^17^, Seth Perlman^6^, Leoyklang Petcharet^4,5^, Lorraine Potocki^11^, Carlos Prada^29^, Rakale C. Quarells^37^, Aaron Quinlan^8^, Daniel J. Rader^38^, Ramakrishnan Rajagopalan^21^, Deepak A. Rao^20^, Anna Raper^38^, Wendy Raskind^6^, Adriana Rebelo^12^, Kelly Regan-Fendt^29^, Chloe M. Reuter^7^, Lynette Rives^17^, Matthew Robinson^10^, Lance H. Rodan^20^, Martin Rodriguez^23^, Mar´ıa Jos´e Ortun˜o Romero^14^, Jill A. Rosenfeld^11^, Elisabeth Rosenthal^6^, Francis Rossignol^4,5^, Bianca E. Russell^24^, Marla Sabaii^4,5^, Mohamad Saifeddine^19^, Jacinda B. Sampson^7^, Suzanne Sandmeyer^3^, Dana Sayer^39^, Timothy Schedl^13^, Jason Schend^19^, Lisa Schimmenti^28^, Kelly Schoch^36^, Jennifer Schymick^7^, Daryl A. Scott^11^, Teodoro Jerves Serrano^14^, Elaine Seto^11^, Mariya Shadrina^15^, Vandana Shashi^36^, Emily Shelkowitz^6^, Susan Shin^15^, Jimann Shin^13^, Saskia Shuman^15^, Cathy Shyr^17^, Mar´ıa Paula Silva^29^, Edwin K. Silverman^20^, Giorgio Sirugo^38^, Kathy Sisco^30^, Tammi Skelton^23^, Cara Skraban^21^, Anne Slavotinek^33^, Carson A. Smith^12^, Kevin S. Smith^7^, Jared Sninsky^11^, Nara Sobreira ^10^, Lilianna Solnica-Krezel^13^, Ben Solomon^4,5^, Albert R. La Spada^3^, Michele Spencer-Manzon^14^, Rebecca C. Spillmann^36^, Maija-Rikka Steenari^3^, Andrew Stergachis^6^, Joan M. Stoler^20^, Kathleen Sullivan^21^, Shamil R. Sunyaev^32^, David A. Sweetser^20^, Barbara N. Pusey Swerdzewski^4^, Virginia Sybert^6^, Holly K. Tabor^7^, Queenie Tan^28^, Arjun Tarakad^11^, Herman Taylor^37^, Mustafa Tekin^12^, Willa Thorson^12^, Cynthia J. Tifft^4,5^, Winston Timp^10^, Camilo Toro^4^, Jennifer Tousseau^39^, Alyssa A. Tran^11^, Kayla M. Treat^27^, Brianna Tucker^7^, Rachel A.Ungar^7^, Filippo Pinto e Vairo^28^, Adeline Vanderver^21^, Andres Vargas^24^, Vasilis Vasiliou^14^, Matt Velinder^8^, James Verbsky^16^, Francesco Vetrini^27^, Eric Vilain^3^, Dave Viskochil^8^, Tiphanie P. Vogel^11^, Colleen E. Wahl^4^, Melissa Walker^20^, Nicole M. Walley^36^, Jennifer Wambach^30^, Emily Wang^14^, Michael F. Wangler^22^, Patricia A. Ward^31^, Isum Ward^19^, Alistair Ward^8^, Stephanie M. Ware^27^, Teneasha Washington^23^, Daniel Wegner^30^, Corrine K. Welt^8^, Mark Wener^6^, Monte Westerfield^41^, Matthew T. Wheeler^7^, Jordan Whitlock^25^, Laurens Wiel^7^, Brandon M. Wilk^23^, Philip Dane Witmer^10^, Elizabeth Wohler^10^, Lynne A. Wolfe^4,5^, Heidi Wood^4,5^, Kim Worley^11^, Elizabeth A. Worthey^23^, Changrui Xiao^3^, Hua Xu^14^, Shinya Yamamoto^22^, Kai Lee Yap^29^, Stephan Zu¨chner^12^, Hui Zhang^14^, Michael T. Zimmermann^16^

*^3^ University of California Irvine (UCI)/Children’s Hospital of Orange County (CHOC): Clinical Site*

*^4^ National Institutes of Health (NIH) Undiagnosed Diseases Program (UDP): Clinical Site*

*^5^ National Institutes of Health (NIH) National Human Genome Research Institute (NHGRI)*

*^6^ University of Washington (UW) Medical Center/Seattle Children’s Hospital: Clinical Site*

*^7^ Stanford University, Center for Undiagnosed Diseases: Clinical Site*

*^8^ University of Utah Health: Clinical Site*

*^9^ Stanford University: Data Management and Coordinating Center*

*^10^ Johns Hopkins Medicine/Kennedy Krieger Institute: Clinical Site*

*^11^ Baylor College of Medicine: Clinical Site*

*^12^ University of Miami: Clinical Site*

*^13^ Washington University at Saint Louis: Model Organisms Screening Center*

*^14^ Yale University: Clinical Site*

*^15^ Mount Sinai: Clinical Site*

*^16^ Medical College of Wisconsin, Central Washington: Clinical Site*

*^17^ Vanderbilt University Medical Center: Clinical Site*

*^18^ Associated Regional and University Pathologists, Inc. (ARUP Laboratories) at the University of Utah: Clinical Site*

*^19^ Sanford Health: Clinical Site*

*^20^ Harvard-affiliated hospitals Boston Children’s Hospital, Brigham and Women’s Hospital, and Massachusetts General Hospital: Clinical Site*

*^21^ Children’s Hospital of Philadelphia (CHOP): Clinical Site*

*^22^ Baylor College of Medicine: Model Organisms Screening Center*

*^23^ University of Alabama at Birmingham Medicine: Clinical Site*

*^24^ University of California at Los Angeles: Clinical Site*

*^25^ University of Alabama at Birmingham: Data Management and Coordinating Center*

*^26^ Washington University at Saint Louis: Data Management and Coordinating Center*

*^27^ Iowa University: Clinical Site*

*^28^ Mayo Clinic: Clinical Site*

*^29^ Lurie Children’s Hospital: Clinical Site*

*^30^ Washington University at Saint Louis: Clinical Site*

*^31^ Baylor College of Medicine: Sequencing Core*

*^32^ Harvard Medical School: Data Management and Coordinating Center*

*^33^ Cincinnati Children’s Hospital: Clinical Site*

*^34^ New York Genome Center: Clinical Site*

*^35^ University of Utah: Data Management and Coordinating Center*

*^36^ Duke University: Clinical Site*

*^37^ Morehouse: Data Management and Coordinating Center*

*^38^ Children’s Hospital of Philadelphia (CHOP) and University of Pennsylvania: Clinical Site*

*^39^ Undiagnosed Diseases Network Foundation*

*^40^ University of Oregon: Model Organisms Screening Center*
